# Minerval (2-hydroxyoleic acid) causes cancer cell selective toxicity by uncoupling oxidative phosphorylation and compromising bioenergetic compensation capacity

**DOI:** 10.1042/BSR20181661

**Published:** 2019-01-18

**Authors:** Wessal Massalha, Mark Markovits, Edward Pichinuk, Yael Feinstein-Rotkopf, Mark Tarshish, Kumudesh Mishra, Victoria Llado, Miguel Weil, Pablo V. Escriba, Or Kakhlon

**Affiliations:** 1 Department of Neurology, Hadassah-Hebrew University Medical Center, Ein Kerem, 91120 Jerusalem, Israel; 2 Laboratory for Neurodegenerative Diseases and Personalized Medicine, Department of Cell Research and Immunology, The George S. Wise Faculty for Life Sciences, Sagol School of Neurosciences, Tel Aviv University, Ramat Aviv, 69978 Tel Aviv, Israel; 3 Light Microscopy Lab, Core Research Facility, Faculty of Medicine, The Hebrew University of Jerusalem, Ein Kerem, 91120 Jerusalem, Israel; 4 Laboratory of Molecular Cell Biomedicine, Department of Biology, University of the Balearic Islands, Ctra. de Valldemossa km 7.5, E-07122 Palma de Mallorca, Spain

**Keywords:** cancer, glycolysis, membrane lipid therapy, Minerval (2-hydroxyoleic acid), mitochondria, oxidative phosphorylation

## Abstract

This work tests bioenergetic and cell-biological implications of the synthetic fatty acid Minerval (2-hydroxyoleic acid), previously demonstrated to act by activation of sphingomyelin synthase in the plasma membrane (PM) and lowering of phosphatidylethanolamine (PE) and phosphatidylcholine (PC) and their carcinogenic signaling. We show here that Minerval also acts, selectively in cancer cell lines, as an ATP depleting uncoupler of mitochondrial oxidative phosphorylation (OxPhos). As a function of its exposure time, Minerval compromised the capacity of glioblastoma U87-MG cells to compensate for aberrant respiration by up-modulation of glycolysis. This effect was not exposure time-dependent in the lung carcinoma A549 cell line, which was more sensitive to Minerval. Compared with OxPhos inhibitors FCCP (uncoupler), rotenone (electron transfer inhibitor), and oligomycin (F1F0-ATPase inhibitor), Minerval action was similar only to that of FCCP. This similarity was manifested by mitochondrial membrane potential (MMP) depolarization, facilitation of oxygen consumption rate (OCR), restriction of mitochondrial and cellular reactive oxygen species (ROS) generation and mitochondrial fragmentation. Additionally, compared with other OxPhos inhibitors, Minerval uniquely induced ER stress in cancer cell lines. These new modes of action for Minerval, capitalizing on the high fatty acid requirements of cancer cells, can potentially enhance its cancer-selective toxicity and improve its therapeutic capacity.

## Introduction

α-Hydroxy-9-cis-octadecanoic acid (2OHOA, Minerval) represents a new class of orally bioavailable lipids and is considered a nontoxic synthetic derivative of oleic acid that was designed to treat human cancer [1]. Minerval can impair proliferation by arresting cell cycle progress from G1 to S phase and has anti-neoplastic effects in cell and animal models of cancer [1–3]. Its mechanism is based on the regulation of the membrane lipid structure. This structural change causes changes in expression, localization, and activity of proteins such as the anti-proliferative protein, protein kinase C (PKC), activated by Minerval [4], the cell proliferation inducer E2F-1, repressed by Minerval [5], and the Ras oncogene translocated from the PM to the cytoplasm, and thus inactivated, by Minerval [6].

Anti-cancer drugs such as Minerval, which interact with membrane lipids and which modify the composition and structure of cell membranes, belong to a conceptually new approach for treating cancer called membrane lipid therapy (MLT) [7]. Minerval is a good example for an MLT drug as it increases the non-lamellar propensity of membrane lipids, activating PKC. In addition, Minerval reduces phospholipid surface packing at the interface between proteins and the polar regions of the lipid bilayer. This increased and enhanced fluidity allows hydrophobic domains of peripheral proteins to interact with deep hydrophobic regions of the membrane and/or fatty acid moieties of phospholipids which protrude out of the bilayer plane [8]. This is the mode of action by which Minerval anchors PKC to the plasma membrane and thus activates it [6,7,9].

In addition to modulation of the PM, Minerval also specifically modulates the activity of sphingomyelin synthase (SMS1), leading to an increased concentration of sphingomyelin (SM) in cancer cells but not in non-cancer cells, in which SM levels are already high [10]. These changes appear to be essential for the anti-proliferative effect of Minerval in glioma cells. In addition, Minerval can increase the levels of diacylglycerol (DAG), reduce the levels of oleic acid, which it can replace [11], and increase the amount of saturated fatty acids incorporated in ordered PC and PE domains in a number of cancer cells, in which membrane fluidity is relatively lower, but not in non-cancer cells. This increase in FA saturation in ordered domains increased their separation from the rest of the membrane thus reducing overall order and liquefying the membrane. Minerval mediated this local increase in saturation by inhibiting stearoyl-CoA desaturase (SCD1) activity, an enzyme that catalyzes the rate-limiting step in the synthesis of unsaturated fatty acids [10–12].

Due to the promising anti-cancer effect of Minerval in lung and leukemic cancer cells and its lipid nature, it was decided to explore it as a treatment against glioma in a phase IIb clinical trial currently in progress (European Commission H2020-SC1-2017-Two-Stage-RTD: A Clinical Phase IIB trial with Minerval in patients with newly diagnosed malignant glioma). This decision is based on the demonstration that Minerval has greater efficacy than the first-line standard of care temozolomide. It is expected that concurrent treatment with both will increase the patients’ progression-free and overall survivals. Moreover, in contrast with temozolomide, there is no apparent relapse in the tumor formation after treatment with Minerval [1,2,13,14].

As Minerval is known to increase membrane fluidity and to be selectively toxic to cancer cells, we set out here to explore the possibility that Minerval, like other hydroxylated fatty acids [15,16], can depolarize mitochondrial inner membranes and thus act as an OxPhos uncoupler selective to cancer cells. We tested the effect of Minerval, in cancer and non-cancer cell lines, on the bioenergetic parameters that should be modified by an uncoupler: Basal oxygen consumption rate (OCR), ATP levels, proton leak, coupling efficiency, maximal respiration, and spare respiratory capacity. Moreover, we have also tested, in cancer and non-cancer cell lines, the effect of Minerval on the ability to respond to stress by up-modulating alternative energy transformation pathways. We have compared Minerval with other established OxPhos inhibitors in terms of its effects on MMP, ROS production, mitochondrial fragmentation, and changes in other cell features manifested as morphological modifications in different organelles. Our results suggest that Minerval functions as a cancer-selective OxPhos uncoupler with the added toxic value of compromising glycolytic stress response and enhancement of ER stress.

## Experimental

### Materials

Glucose, paraformaldehyde (PFA) and sodium pyruvate were procured from Sigma. DMEM (with and without phenol red (for microscopy)), RPMI and EMEM media, as well as L-Glutamine, cell culture antibiotics (penicillin–streptomycin–neomycin), fetal calf sera, trypsin, trypan blue (for live cell counting in seeding), and phosphate buffered saline (Ca and Mg free) were all procured from Biological Industries (Beit HaEmek, Israel). CellROX Green, MitoTracker Red (CMXRos), MitoSox red, ER-Tracker red, LysoTracker Deep Red, DAPI, Calcein-AM Green, and TMRE (tetramethylrhodamine, ethyl ester) were all purchased from Thermo Fisher Scientific.

### Cells

We used three human cell lines: primary glioblastoma (U87-MG), non-small cell lung adenocarcinoma (A549), and non-cancerous fibroblast-like MRC5 cells derived from fetal lung. U87-MG cells were grown in DMEM medium supplemented with 4.5 g/l glucose, 10% fetal bovine serum and 1 mM L-glutamine, sodium pyruvate, and penicillin–streptomycin–neomycin. A549 and MRC5 cells were respectively grown in RPMI and EMEM media with the same supplements.

### Bioenergetic assays

Bioenergetic assays (parallel monitoring of respiratory and glycolytic parameters) were performed using the XF Cell Mito Stress Test Kit (Agilent, Seahorse) following manufacturer’s instructions. These measurements are based on extracellular monitoring of oxygen and proton fluxes by fluorescence-based probes. Specifically, for our experiments, cells were seeded in XF96 cell culture plates at 40,000 cells/well. Minerval (final concentration 200 µM [1,3,5,10,12,17]) was added for 24, 48, and 72 h [1,3,5,10,12], OxPhos inhibitors were added in the following final concentrations: oligomycin (ATPase inhibitor), 1 µM; FCCP (Carbonyl cyanide-p-trifluoromethoxyphenylhydrazone, a protonophore), 0.4 µM for U87-MG cells, and 1 µM for A549 and MRC5 cells; Antimycin A and Rotenone (complexes III and I inhibitors, respectively), 1 µM. ATP levels were determined by the CellTiter Glo kit (Promega).

### Confocal microscopy

Confocal studies were performed in the LSM 710 microscope (Zeiss) using a 60/1.35 NA PlanApochromat oil-immersion lens. The ImageJ software was used for analysis and quantification of the fluorescence intensity of the confocal images. To permit comparison of images, the same laser power and detector gain and offset were used for all compared images. The following fluorophores were used: The CellROX® Green Reagent (excitation 488, emission 520 nm; 5 µM in DMEM without phenol red for 30 min, followed by live imaging) to measure overall cellular reactive oxygen species (ROS) production; TMRE (excitation, 543 nm, emission, 633 nm; 500 µM in DMEM without phenol red). In FCCP treatments, TMRE was added to the FCCP containing medium after 30 min without washing). TMRE readily accumulates in active mitochondria due to their relative negative charge. However, TMRE is poorly retained in fixed cells and was therefore only used by us to detect MMP dynamically in live cells. For fixed cells, we used the CMXRos (MitoTracker red) stain (excitation, 543 nm, emission, 633 nm; 500 nM in DMEM without phenol red for 30 min, followed by wash and PFA fixation). MitoSox (excitation, 543 nm, emission, 633 nm; 5 µM in DMEM without phenol red for 30 min before imaging), which is a mitochondrion-targeted dihydroethidium, was used to detect superoxide production (mitochondrial ROS) *in situ* in mitochondria of live cells. Mitochondrial size and fragmentation were determined by the Mito-Morphology macro added as a plugin to the ImgaeJ image analysis software. We analyzed our confocal microscope images using this macro.

### Multiparameter analysis

U87-MG, MRC5, and A549 cells were cultured in specialized microscopy-grade 96-well plates (Grenier Bio-One, GER). Minerval (200 μM) dissolved in DMSO was added only for 72 h, in order to maximize its effects. Total DMSO concentrations were always kept below 0.1%. After a process of optimization, taking into account the growth of cells during the 72 h period of Minerval (200 µM) exposure, U87-MG and A549 cells were seeded at a concentration of 800 per well, and MRC5 at a concentration of 15,000 per well. In experiments where the effects of Minerval were compared with those of OxPhos inhibitors, these inhibitors were added to cells, pre-treated for 72 h with DMSO as a vehicle control, for 30 min in the same concentrations specified in Bioenergetic Assays. Following the 72 h Minerval/control treatments, a mix of cellular fluorescent dyes in PBS was added to each well for 30 min at 37°C in a 5% CO_2_ incubator. This mix included ER-Tracker red (1 μM, an endoplasmic reticulum (ER) stain), LysoTracker Deep Red (75 nM, a lysosome stain), DAPI (1:10,000, nuclear (DNA) stain), and Calcein-AM Green (10 μM, a cytosol stain used for cell segmentation). Cells were then fixed with 4% paraformaldehyde (PFA), washed with PBS and plates were transferred to an InCell2200 (GE Healthcare, U.K.) machine for image acquisition at 40× magnification. The output produced was based on comparative fluorescence intensity. Object segmentation was carried out using Multi-target analysis in the GE analysis workstation to identify the nuclei (DAPI) and cell boundary (Calcein Green). We further identified ER (ER-Tracker) and lysosomes (LysoTracker) as intracellular ‘organelle’ objects. All the assay parameters (including the acquisition exposure times, objective, and the analysis parameters) were kept constant for all assay repetitions.

## Results

### Bioenergetic effects of Minerval

**Figure 1:**
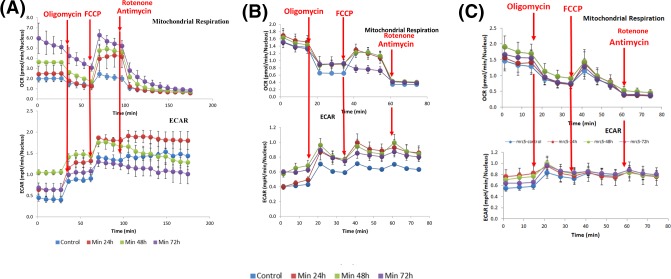
The effect of Minerval on bioenergetic profiles Cell bioenergetics (OCR, oxygen consumption rate, and ECAR, extracellular acidification rate) in U87-MG (**A**), A549 (**B**), and MRC5 (**C**) cells treated with Minerval for different periods of time as indicated were analyzed by the Agilent’s Seahorse machine, as described in ‘Experimental’ section. Compounds added where indicated. OCR and ECAR are expressed per nucleus. A representative experiment out of *n*=3 performed is shown for each cell line. Details are provided in text.

**Figure 2:**
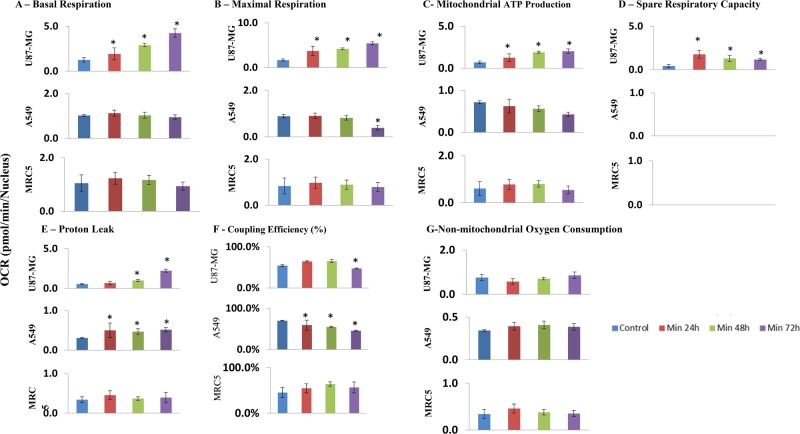
Bioenergetic parameters in the U87-MG, A549, and MRC5 cell lines (**A**) Basal respiration was averaged from the initiation of the experiment until the addition of oligomycin. (**B**) Maximal respiration was averaged from the addition of FCCP to the addition of rotenone+antimycin A. (**C**) ATP production is defined as the average difference (basal OCR – OCR after oligomycin). (**D**) Spare respiratory capacity is defined as the average difference (maximal OCR − basal OCR). (**E**) Proton leak is the average OCR measured from the addition of oligomycin to the addition of FCCP. (**F**) Coupling efficiency is defined as the average quotient (OCR following Oligomycin)/(Basal OCR). (**G**) Non-mitochondrial OCR reflects the oxygen consumption that persists after mitochondrial respiration is blocked by antimycin+rotenone. Values significantly different from Control (*P*<0.05), as determined by One-Way ANOVA with Dunnett post-hoc test, are denoted by an asterisk.

The inhibitors oligomycin, FCCP, and rotenone plus Antimycin A were sequentially injected by the Seahorse XF machine to measure OCR driving ATP production, maximal respiration, and non-mitochondrial respiration, respectively. In parallel, the extracellular acidification rate (ECAR) was also measured in response to these inhibitors. These Seahorse bioenergetic profiling experiments were applied to the U87-MG (glioblastoma), A549 (lung adenocarcinoma) and, for comparison, MRC5 (non-cancerous) cell lines. Minerval at 200 µM was added for 24, 48, and 72 h to all cell lines. The choice of these times and concentration is based on previous work in these cell lines showing time-escalation of various cancer growth-diminishing parameters [3,5,10,12,18]. Figure 1 shows the raw results of these Seahorse XF bioenergetic assays. These results are quantified in Figure 2.

### Tissue of origin-based comparison of the bioenergetic effects of Minerval

In the glioblastoma U87-MG cells, basal OCR (Figure 2A) and maximal respiration (Figure 2B), probably in consequence of the elevated baseline, are significantly increased by Minerval, especially following 48 and 72 h exposure. This increase in basal OCR, in U87-MG, but not in A549 and MRC5 cells (Figure 2A), explains the relatively high mitochondrial ATP production (basal OCR – OCR after oligomycin) instigated by Minerval only in U87-MG cells (Figure 2C). As the Minerval-dependent increase in basal OCR levels shows (Figure 2A), U87-MG cells progressively increase their energy demand [19] as time of exposure to Minerval increases. This increase in energy demand, observed only in U87-MG and not in A549 and MRC5 cells, possibly explains the U87-MG cell-specific up-modulation of mitochondrial ATP production (Figure 2C), which could be a U87-MG-specific pre-requisite for this cell line survival in the presence of Minerval.

In contrast with U87-MG cells, Minerval did not significantly affect basal (Figure 2A) and maximal (Figure 2B) OCR in both A549 and MRC5 lung cell lines, except for maximal OCR after 72 h exposure in A549 cells (Figure 2B), where the Minerval–FCCP combination was possibly too toxic, or reduced mitochondrial mass (see also Figure 1B). These observations can be explained in terms of the spare respiratory capacity (maximal OCR – basal OCR, Figure 2D): A549 and MRC5 cells have no spare respiratory capacity (basal OCR and OCR following FCCP addition are not significantly different, Figure 1B,C, upper panels). This observed lack of spare respiratory capacity in A549 and MRC5 cells, also corroborated in the literature [20,21], means that already at basal conditions, mitochondrial respiration generates ATP energy at its full capacity. Therefore, as opposed to U87-MG cells that do have a spare respiratory capacity (Figure 2D), no significant effect of Minerval on the already maximal basal OCR in A549 and MRC5 cells was observed (Figure 2A). Interestingly, the spare respiratory capacity in U87-MG cells was most up-modulated by Minerval after 24 h (and not 72 h) of exposure, possibly reflecting the relatively more pronounced effect of longer exposures to Minerval on the basal OCR (Figure 2A).

### Cancer-selective bioenergetic effects of Minerval

Minerval-triggered increases in proton leak (Figure 2E) and consequent decreases in coupling efficiency (Oligomycin OCR/Basal OCR, Figure 2F) were distinctively observed in the cancerous U87-MG and A549 cell lines and not in non-cancerous MRC5 cells. These changes indicate that Minerval acted as a partial OxPhos uncoupler, which, similar to UCP2 overexpression for instance [22], increases basal OCR and proton leak, but still preserves OCR sensitivity to oligomycin and leaves room for induction of maximal respiration by an adequate dose of the stronger uncoupler FCCP. Compensatory glycolysis was also stimulated only in the U87-MG and A549 cell lines and not in the MRC5 cell line (compare Figure 1 A,B with Figure 1C (lower panels)). However, this effect was qualitatively different between the two cancer cell lines: in the A549 cells, glycolysis was stimulated to the same extent independently of Minerval exposure time (Figure 1B, lower panel). On the other hand, in U87-MG cells, glycolytic compensation correlated with Minerval exposure time. As shown in Figure 1A (lower panel), in U87-MG cells the capacity to compensate for deficient respiration by up-modulation of ECAR is gradually compromised as Minerval exposure time increases: Following 24 h exposure to Minerval, U87-MG cells are able to up-regulate glycolysis (ECAR) after oligomycin, FCCP and antimycin A/rotenone are added. Following 48 h exposure, they are only able to compensate (up-regulate ECAR) for oligomycin and FCCP, and following 72 h exposure, they are only able to compensate for oligomycin, and even that to a lesser extent than after 24 and 48 h Minerval exposure. Interestingly, in U87-MG cells glycolytic compensation capacity is inversely related to the energy demand (basal OCR), which is increased by Minerval in these cells. Notably, non-mitochondrial OCR (Figure 2G) was not affected by Minerval in all cell lines.

**Figure 3:**
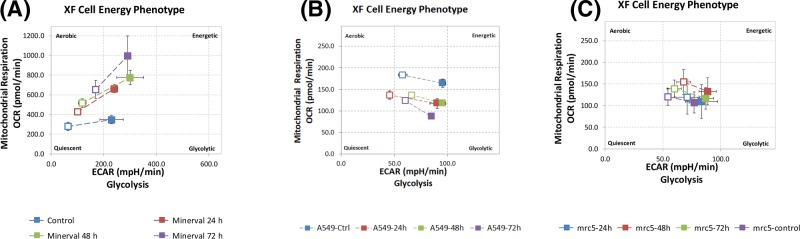
The effect of Minerval on energy profiles Energy profile in U87-MG (**A**), A549 (**B**), and MRC5 (**C**) cells exposed to 200 µM Minerval for the durations indicated. Shown are OCR and ECAR responses to FCCP stress. Empty squares, baseline OCR, and ECAR values; Filled squares, FCCP stressed OCR, and ECAR values.

The energy diagrams in Figure 3 reflect both respiratory (*y*-axis) and glycolytic (*x*-axis) contributions to overall cell energetic status, both at basal (empty squares) and maximal (filled squares) respiration. According to these diagrams, Minerval treatment rendered U87-MG cells more energetic, or closer to the upper right-hand corner of the energy diagram (Figure 3A), while it decreased energy status in A549 cells (Figure 3B) and did not significantly modify the energy status in MRC5 cells (Figure 3C). These Minerval-caused changes apply to both basal and maximal respiration states. The inhibitory effect of Minerval exposure on glycolytic compensation capacity is demonstrated by the increase in the OCR/ECAR slope with time of exposure to Minerval, especially at 72 h (Figure 3A). Overall, the increase in energy status only in U87-MG cells supports the conclusion that Minerval was more toxic to A549 cells than it was to the U87-MG cells, while not affecting the non-cancerous MRC5 cells.

### Minerval effect on metabolic cell viability

**Figure 4:**
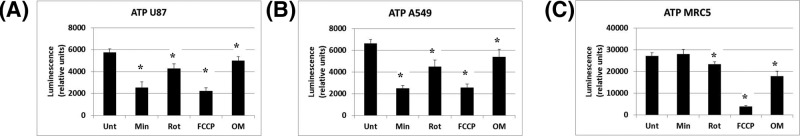
The effect of Minerval, compared with mitochondrial inhibitors, on ATP/viability (the extent of metabolically viable cells) ATP, or the extent of metabolically viable cells, was determined in the indicated cell lines (**A**) U87-MG, (**B**) A549, and (**C**) MRC5 untreated or treated with Minerval for 72 h (Min), with 1 μM rotenone for 30 min (Rot), with FCCP (10 μM for U87-MG cells, 1 μM for A549 and MRC5 cells) for 30 min (FCCP), or with 1 μM oligomycin for 30 min (OM). Values significantly different from Control (*P*<0.05), as determined by One-Way ANOVA with Dunnett post-hoc test, are denoted by asterisks.

The effect of Minerval on cell viability and/or ATP levels (Figure 4) brings together all its bioenergetic and metabolic effects. Overall ATP level is the ultimate co-reporter of both viability and energetic status. Reduced ATP level is a rigorous indication for cellular stress. Therefore, in order to determine whether the bioenergetic effects described above can significantly influence the cellular stress response to Minerval, we decided to analyze overall ATP levels. Our results (Figure 4A,B) show that while the energetic status of U87-MG cells was increased (Figure 3A), and while compensatory glycolysis was induced in a Minerval-independent manner in A549 cells (Figure 1B), these changes were futile inasmuch as they were not coupled to increased metabolic viability or ATP production in both cancer cell lines. In fact, Minerval at 72 h has reduced metabolic viability in U87-MG and A549 cells to an extent unmatched by the OxPhos inhibitors rotenone and oligomycin and only matched by the uncoupler FCCP (Figure 4A,B). The slightly larger drop in ATP elicited by Minerval in A549 cells (37% of control, Figure 4B), as compared with U87-MG cells (44% of control, Figure 4A) may also point to higher toxicity of Minerval toward the former. As opposed to the U87-MG and A549 cancer cell lines, no bioenergetic effect of Minerval was observed in the non-cancerous cell line MRC-5 (Figures 1C, 2, and 3C). Accordingly, MRC5 cells demonstrated no significant response to Minerval in terms of metabolic viability (as opposed to their response to the other inhibitors, Figure 4C).

In summary, our work demonstrates for the first time that the well documented [2,3,10–12,23] cancer-selective sensitivity to Minerval can also be manifested by mitochondrial and cell energy functions.

### Minerval compared with an OxPhos uncoupler in U87-MG cells

**Figure 5:**
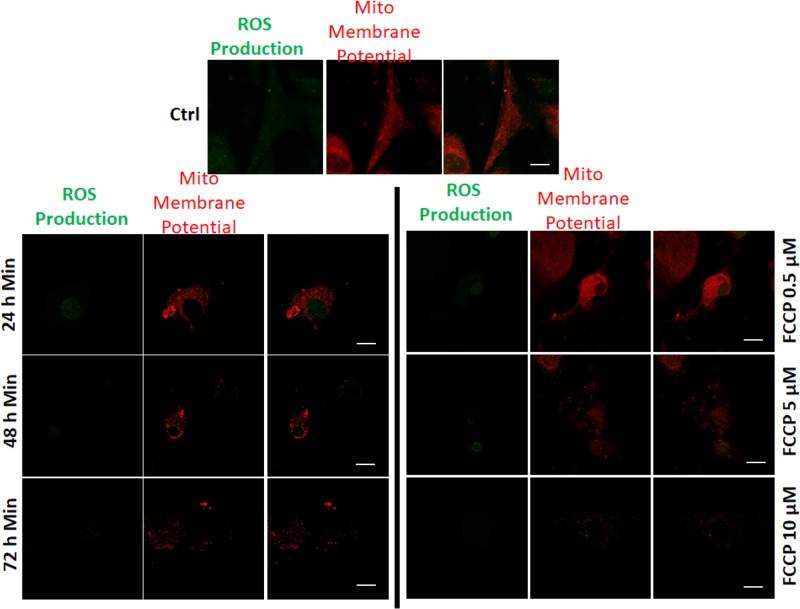
The effect of Minerval, compared with an uncoupler, on cellular ROS production in U87-MG cells U87-MG cells were untreated or treated in parallel with 200 µM Minerval for the indicated durations or for FCCP in the indicated concentrations. MMP and overall cellular ROS production were detected by staining with TMRE and CellROX, respectively and confocal microscope analysis as described in ‘Experimental’ section was conducted; scale bars, 10 μm.

The Minerval-mediated increase in proton leak and decrease in coupling efficiency observed in cancer cells indicate that in these cells the drug acts as an OxPhos uncoupler. We therefore decided to compare the effects of Minerval with those of the established OxPhos uncoupler FCCP. We opted to conduct this comparison in the glioblastoma cell line U87-MG. The justification for this decision is the following: as an anticancer drug, Minerval was so far tested only in glioblastoma patients. In addition, the glioblastoma cell line U87-MG is relatively less sensitive to Minerval (Figures 3 and 4) and so has more room for mechanistically based improvement. As compared with other glioma cell lines such as SF767 [1], U87-MG cells generate more pronounced xenograft tumors that are also more responsive to Minerval (VL and PVE, unpublished results). Therefore, we conducted an in-depth comparison between Minerval and FCCP in U87-MG cells as a model for cancer. First we have demonstrated the dose response of the Minerval effect. As Figure 5 shows, longer exposures to Minerval (at 200 µM) correlated with MMP depolarization and with attenuation of cellular ROS production. Minerval exposure time was equivalent to increase in FCCP concentration which produced similar effects on both MMP and cellular ROS production.

**Figure 6:**
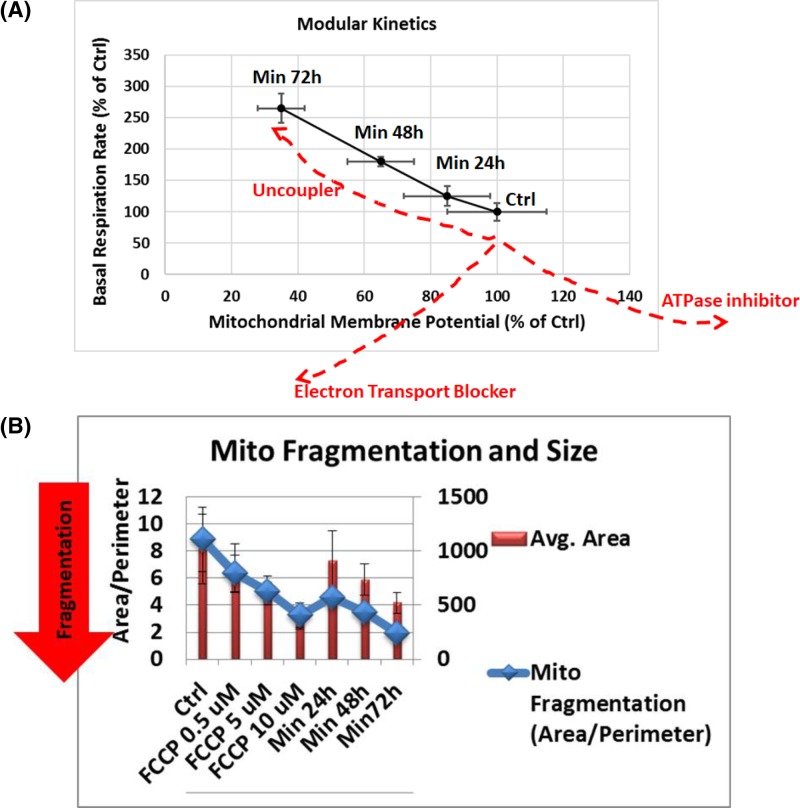
The effects of Minerval on modular kinetics and mitochondrial morphology in U87-MG cells (**A**) U87-MG cells were treated with vehicle DMSO control or exposed to 200 µM Minerval for the indicated durations and then were split to Seahorse and confocal microscope analysis in order to determine in parallel basal OCR and MMP (modular kinetics chart). Means and standard deviations of OCR and MMP values based on *n* = 3 experiments are presented as percentages of control. Dashed red lines show putative effects of the indicated OxPhos inhibitors. (**B**) The effect of Minerval exposure time and FCCP levels on the size, or area of solitary mitochondria (red bars) and on the extent of fragmentation from the mitochondrial network (blue diamonds) as determined by the area to perimeter ratio. Confocal images were analyzed in U87-MG cells by the Mito-Morphology macro as described in ‘Experimental’ section.

We have further confirmed the similarity between Minerval and FCCP by modular kinetics and mitochondrial dynamics. Figure 6A shows MMP and basal OCR values measured in the same batch of cells that were analyzed in parallel by the Seahorse machine (for OCR measurements) and by confocal microscopy (for MMP quantification). Treatment with an OxPhos uncoupler is expected to increase OCR as MMP is depolarized because this depolarization facilitates electron flow down the respiratory chain. By contrast, an electron transport chain (ETC) or respiratory complex blocker, such as rotenone which blocks complex I, would reduce OCR in parallel to depolarizing MMP, since it attenuates ETC flow. H^+^-ATPase blocker, such as oligomycin, on the other hand, would be expected to dose-responsively increase MMP as protons are prevented from re-entering from the inter-membrane space into the mitochondrial matrix and thus dissipating MMP. This block in MMP dissipation and higher MMP would hinder ETC flow that is coupled with proton extrusion from the matrix to the inter-membrane space. Therefore, as H^+^-ATPase inhibition gradually increase, OCR would decrease. Therefore, as can be observed in Figure 6A, our modular kinetics data prove that Minerval has a mode of action identical with an OxPhos uncoupler.

Figure 6B describes the effect of Minerval on mitochondrial dynamics. Mitochondrial dynamics (the extent to which mitochondria are connected in a network or fragmented) determines energy expenditure and nutrient utilization [24]. These parameters are paramount to cancer cells proliferation capacity and invasiveness. As our results (Figure 6B) show, Minerval caused mitochondrial fragmentation, assessed by the area to perimeter ratio, and shrinking (area decrease). These effects of Minerval were pheno-copied by the uncoupler FCCP and, similar to the other mitochondrial effects (Figures 5 and 6A), were augmented with Minerval exposure time or with FCCP concentration. These results are consistent with increased OCR due to induced proton conductance, and not inherent proton leak. Induced proton conductance by chemical compounds such as FCCP, or by endogenous uncoupling proteins (UCPs), is associated with fragmentation of the mitochondrial network, through the fission protein Drp 1, into doughnut-shaped mitochondria [24]. These effects of Minerval, and FCCP as a positive control for induced uncoupling, can reduce ATP production efficiency (see also Figure 4). ATP production efficiency can also be reduced by nutrient excess, also associated with increased fragmentation. However, in the latter case, MMP is increased (due to higher substrate availability from nutrients), rather than decreased, and thus also increases the inherent proton leak (positively correlated with MMP). These increased MMP and inherent proton leak are associated with an increase, rather than a decrease, in the diameter of solitary mitochondria [24], possibly because the inner membrane of the mitochondria has to accommodate more MMP determining proteins, mainly the adenine nucleotide translocators [25]. Therefore, the combination of mitochondrial fragmentation together with a decreased, rather than increased, diameter of solitary mitochondria points to a Minerval associated reduction of ATP production efficiency which is caused by induced uncoupling and not by a wasteful nutrient excess, as could possibly be expected from the faster internalization of Minerval in cancer cells [10,11,26] and its conceivable use as fuel. Therefore, Minerval reduces ATP production efficiency like an uncoupler and not by inefficient, heat producing energy transformation typical of cells under nutrient excess conditions. These data corroborate the well documented inability to metabolize Minerval as a lipid nutrient or fuel [6].

### Minerval effect compared with other OxPhos inhibitors

#### Comparative effect of Minerval on MMP

**Figure 7:**
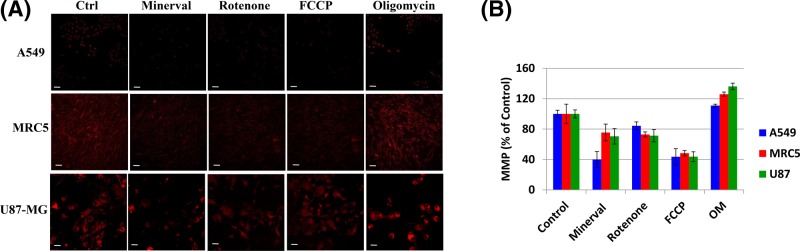
The effect of Minerval, compared with mitochondrial inhibitors, on mitochondrial membrane potential (**A**) A549, MRC5, and U87-MG cell lines, as indicated, were untreated (Ctrl) or treated with Minerval for 72 h (Min), with 1 μM rotenone for 30 min (Rot), with FCCP (10 μM for U87-MG cells, 1 μM for A549 and MRC5 cells) for 30 min (FCCP), or with 1 μM oligomycin for 30 min (Oligomycin). Cells were then washed in PBS (except for FCCP) and stained for 30 min with 500 nM CMXRos, fixed in 4% PFA and visualized by the Zeiss LSM confocal microscope. Acquisition settings among the different treatments were identical. (**B**) Fluorescence intensity in (A) quantified as described in ‘Experimental’ section and expressed as percentage of control. Bars show means and standard deviations of *n*=4 independent experiments. All MMP values were significantly different from Control (*P*<0.05), as determined by One-Way ANOVA with Dunnett post-hoc test. Scale bars are 100 µm for A549 and MRC5 cells and 20 µm for U87-MG cells.

Having established that Minerval acts as a partial uncoupler, we decided to confirm these results microscopically by comparing the effect of Minerval to that of established mitochondrial inhibitors. Figure 7A (quantified in Figure 7B) shows that, similar to FCCP and to rotenone, administration of Minerval led to depolarization of the MMP. This effect was observed in both cancerous (A549, U87-MG) and non-cancerous (MRC5) cell lines. Expectedly, and also as a positive control for the ability to detect MMP modifications, MMP was hyperpolarized by the H^+^-ATPase blocker oligomycin (OM), which restricts proton flow through the complex down its electrochemical gradient.

#### Comparative effect of Minerval on mitochondrial ROS production

**Figure 8:**
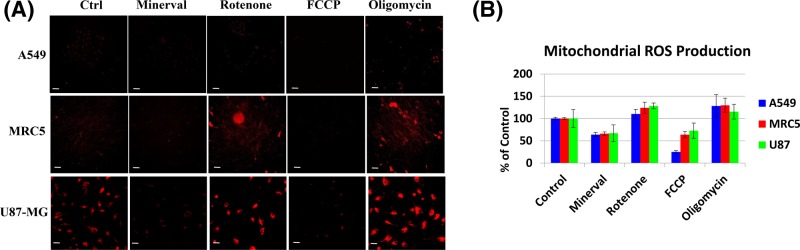
The effect of Minerval, compared with mitochondrial inhibitors, on mitochondrial ROS production (**A**) A549, MRC5, and U87-MG cell lines, as indicated, were treated as described in Figure 5. Cells were then stained with 5 µM MitoSOX, washed in PBS (except for FCCP) and visualized by the Zeiss LSM confocal microscope. Acquisition settings among the different treatments were identical. (**B**) Fluorescence intensity in (A) was quantified as described in ‘Experimental’ section and is expressed as percentage of control. Bars show means and standard deviations of *n*=4 independent experiments. All mitochondrial ROS production values were significantly different from Control (*P*<0.05), as determined by One-Way ANOVA with Dunnett post-hoc test. Scale bars are 100 µm for A549 and MRC5 cells and 20 µm for U87-MG cells.

MMP depolarization by the uncoupler FCCP occurs by a mechanism different than that of the respiratory chain blocker rotenone: while FCCP is a protonophore that mitigates MMP by mobilizing protons down their electrochemical gradient, rotenone blocks respiratory complex I thus inhibiting electron flow that generates this gradient. This fundamental mechanistic difference is expressed by the difference in ROS production: while FCCP facilitates electron flow through the respiratory chain thus diminishing premature reduction of dioxygen into superoxide and ROS production, rotenone slows down this electron flow thus enhancing mitochondrial ROS production. Similarly, the H^+^-ATPase blocker OM can prevent proton re-entry through the inner mitochondrial membrane and consequent MMP dissipation and thus also hinders electron flow and generates ROS. These differences among the inhibitors and the observation that, like the uncoupler FCCP, Minerval too mitigates mitochondrial ROS production are shown in Figure 8, where mitochondrial ROS production is reported by the mitochondrion-targeted dihydroethidium derivative MitoSOX.

#### Multiparametric analysis of the effects of Minerval on U87-MG cells

**Figure 9:**
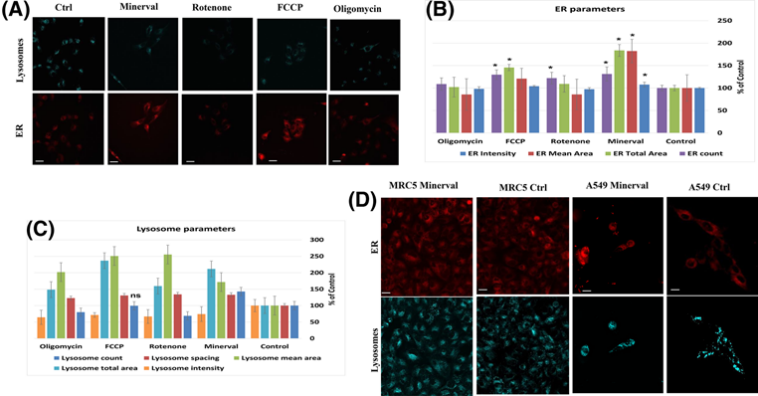
Multiparametric analysis of the effect of Minerval, compared with an uncoupler, in U87-MG cells (**A**) Representative images of ER (red) and lysosomes (blue) obtained by the InCell 2200 high content analysis system following 72 h exposure of U87-MG cells to 200 µM Minerval, or 30 min exposure to 1 µM rotenone, 10 µM FCCP, or 1 µM oligomycin. (**B** and **C**) Bar graphs showing the quantification of the multiparametric effects of Minerval and mitochondrial inhibitors, based on *n*=3 experiments. The indicated ER (B) and lysosome (C) morphological parameters were quantified following the treatments mentioned in (A). Asterisks in (A) denote significant (*P*<0.05) differences from control. Except for lysosomal count in FCCP treated cells all lysosomal parameters in (B) were significantly different than control. Significance was determined by One-Way ANOVA with Dunnett post-hoc test; scale bars, 10 μm. (**D**) The effect of Minerval on ER and lysosomes in cancerous (A549) and non-cancerous (MRC5) cell lines. Representative images of ER (red) and lysosomes (blue) obtained in A549 and MRC5 cells by the InCell 2200 high content analysis system following 72 h exposure to 200 µM Minerval, or to DMSO vehicle control; scale bars, 10 μm.

To obtain a more comprehensive picture of the influence of Minerval at the cell level, we have analyzed and quantified in U87-MG cells the effect of the lipid on cellular features other than the mitochondria using the unique high content, multi-parametric image analyzer InCell 2200 (see ‘Experimental’). Interestingly, Minerval has increased the area, vesicle number and staining intensity of the endoplasmic reticulum (ER) in U87-MG cells (Figure 9). This effect was significantly more pronounced following Minerval treatment, as compared with the other uncoupler FCCP, and was not observed when other OxPhos inhibitors—rotenone and oligomycin—were used (except for a significant increase in ER fragmentation caused by rotenone, Figure 9A,B). These results suggest that Minerval has a unique effect on the ER which goes beyond its effect as an OxPhos uncoupler. Furthermore, the fact that the increase in ER area was considerably higher than the increase in ER intensity suggests that Minerval induced ER dilation. ER dilation and fragmentation suggest that Minerval evoked ER stress, as already shown biochemically in U87-MG cells by up-regulation of unfolded protein response biomarkers [12]. Interestingly, another synthetic lipid evaluated as a cancer therapeutic, N-(4-hydroxyphenyl)retinamide [27] has also dilated the ER. These ER effects may manipulate PE synthesis by the organelle [28] and its transport to the plasma membrane and may therefore have relevance for Minerval gain of toxic function. Furthermore, in our hands too (Figure 9D) this effect on ER stress was unique to cancer cells. Another effect observed by both Minerval and OxPhos perturbators is the increase in the number, area and spacing and decrease in staining intensity of lysosomes (Figure 9A,C). This effect is probably related to the increased cytotoxic autophagy induced by Minerval [12], as the lysotracker lysosomal stain we used can also identify autolysosmes.

## Discussion

### Minerval acts as a partial uncoupler of oxidative phosphorylation

The main finding of this work is that Minerval serves as a partial uncoupler of oxidative phosphorylation (OxPhos). We have shown that, similar to the well-established uncoupler FCCP, Minerval increases proton leak and reduces coupling efficiency in the cancer cell lines U87-MG and A549, while not affecting leak and coupling efficiency in the non-cancerous cell line MRC5. In addition, in U87-MG cells we have demonstrated that the modular kinetics of Minerval correspond only to those of an uncoupling agent, and not to other OxPhos modulators (Figure 6A). Expected bioenergetic effects of different prototypical OxPhos modulators are presented in Table 1, which demonstrates that the bioenergetic effects of Minerval can only match those of an OxPhos uncoupler.

**Table 1:** The influence of different OxPhos modulators on bioenergetic parameters

	Mito. Memb. Potential (CMXRos, potentiometric dye)	Oxygen Consumption Rate (Seahorse)	Reactive Oxygen Species (CellROX)	ATP (CellTiter-GLO)
ETC blocker (rotenone)	↓	↓	↑	↓
OxPhos uncoupler (FCCP)	↓	↑	↓	↓
ATPase blocker (oligomycin)	↑	↓	↑	↓
Substrate oxidation (succinate)	↑	↑	↑	↑
Minerval	↓	↑	↓	↓

Also at the cell biological level Minerval was demonstrated to act as an uncoupler and not as nutrient excess, as the mitochondrial fragmentation it triggered was accompanied by a size decrease, and not increase, of the solitary mitochondria formed by the fragmentation (Figure 6B). Apart from substantiating the clinical advantage of Minerval, as these results point to selective damage to cancer cells only, it was interesting to consider why proton leak was not affected by Minerval in the non-cancerous cell line while it was in the cancer cell lines. Our explanation for this observation is based on the different structure and composition of mitochondrial lipids in cancer cells. Mitochondria of tumor cells are characterized by higher cholesterol levels and saturated fatty acyl chains [29]. These membranes thus tend to be less fluid and consequently less permeable to several substrates including protons (even though for protons the correlation between permeability and fluidity is less tight [30]). The observations that Minerval can increase membrane fluidity [11] and our preliminary lipidomics analysis showing reduction in cholesteryl esters, derived from cholesterol, by Minerval in U87-MG cells (PVE, unpublished results) suggest that Minerval has increased proton conductance selectively in cancer cells. This explanation is in line with the higher cholesterol content and rigidity of mitochondrial membranes in cancer cells that makes them more susceptible to the liquefying effect of Minerval than the mitochondrial membranes of non-cancerous cells such as MRC5. Thus, like FCCP, Minerval is hypothesized to increase proton conductance through the mitochondrial inner membrane. However, while FCCP increases proton conductance in both cancerous and non-cancerous cells by acting as a soluble protonophore, Minerval is hypothesized to selectively increase proton conductance in cancer cells through an increase in inner mitochondrial membrane fluidity. Another explanation, not necessarily mutually exclusive, is that Minerval has been demonstrated to up-regulate the expression of the uncoupling protein UCP1, thus acting as an OxPhos uncoupler [31], even though the tumor selectivity of this effect has not yet been tested.

### MMP depolarization by Minerval and its effect on ROS production

As an MMP depolarizing agent, Minerval is also expected to facilitate electron flux through the respiratory chain and thus reduce mitochondrial ROS production, mainly caused by stalled or back flowing electrons that prematurely reduce dioxygen. This MMP depolarization may be associated with the extent of cancer selective Minerval toxicity, as the largest MMP depolarization was observed in the cancer cell line most susceptible to Minerval, A549 (Figure 7). We speculate that modifications of the biophysical properties of the mitochondrial inner membrane, rather than its lipid composition, are more important for the anti-tumorigenic effect of Minerval. The reason for this speculation is that both isoforms of SMS modulated by Minerval, SMS1 (http://www.uniprot.org/uniprot/Q86VZ5#subcellular_location) and SMS2 (http://www.uniprot.org/uniprot/Q8NHU3#subcellular_location), are not expressed in mitochondrial membranes. Moreover, the modified lipid composition of mitochondria derived from tumors, mainly consisting of reduced levels of mature cardiolipins, is considered irreversible [32]. Possibly, the reduced mature cardiolipins in cancer cell mitochondria increase MMP by decreasing proton conductance through the F0 subunit of the mitochondrial F1F0-ATPase (H^+^-ATPase) [29]. This mode of action is consistent with the higher ROS produced by cancer cells mitochondria. An alternative mode of action involving ETC complexes upstream of F1F0-ATPase is less likely since the observed reduction in ETC complexes I, II, and III [32] associated with tumor cells would decrease rather than increase MMP. Thus, we further speculate that Minerval action counteracts this putative decrease in F0 proton conductance by increasing proton conductance through increasing membrane fluidity. ROS generated by the higher MMP typical of cancer cells can be important for cancer development. For instance, HIF1α-dependent carcinogenesis [33], and, especially important in the context of Minerval, Kras-mediated tumorigenicity [34] are dependent on ROS production. Therefore, the ROS mitigating down-modulation of MMP by Minerval is yet another mechanism discovered by this work which could mediate the attenuation of carcinogenic signaling, in addition to the well-established plasma membrane to cytoplasm translocation of oncoproteins such as Ras.

### Minerval selectivity to cancer cells

Cancer cells have important energetic needs and fatty acids are one of the sources they use to fulfill their growth requirements. Previous studies have shown that intake of the fatty acid analog Minerval is markedly faster in cancer cells with respect to non-tumor cells [11]. Possibly as a consequence of this increased uptake and the slower metabolization of C2-hydroxylated fatty acids, Minerval is cytotoxic toward cancer cells, or tumors, sparing normal cells and normal tissues. This important feature is most pronounced by the effect of Minerval on cell viability and/or ATP levels (Figure 4). The assay we selected for assessing cell viability is the CellTiter-Glo (Promega) that quantifies the number of metabolically active cells by overall ATP levels. This method better serves the cause of assessing selective toxicity because it is based on ATP—the ultimate co-reporter of both viability and energetic status. Other assays, for instance the common 3-(4,5-dimethylthiazol-2-yl)-2,5-diphenyltetrazolium bromide (MTT)-based assays, report the number of metabolically active cells less reliably because they use endogenous NADH and thus divert it from its endogenous intracellular assays, generating metabolic stress. Moreover, the MTT assay postulates that mitochondrial NADH reduces MTT, whereas also NADH stemming from non-mitochondrial or non-energy producing processes, such as endosomal or lysosomal NADH generation, might reduce MTT as false positives.

According to our data, Minerval acted as an uncoupler and reduced MMP in all cell types studied—the cancerous U87-MG and A549 and the non-cancerous MRC5 (Figure 7). However, as expected, Minerval was toxic only to the cancerous cell lines U87-MG and A549 (Figure 4). This result, widely supported by all literature on Minerval in terms of cancer selective abrogation of plasma membrane initiated proliferative signaling, was never explained in bioenergetic terms. We can provide the following bioenergetic explanation: if we compare the cancerous U87-MG cell line (Figure 1A, upper panel) with the non-cancerous MRC5 cell line (Figure 1C, upper panel), we can see that the basal OCR of U87-MG is higher (∼2 pmol O2 reduced/min/nucleus for U87-MG vs 1.5 pmol O_2_ reduced/min/nucleus for MRC5 cells). The higher basal OCR in U87-MG cells suggest they have higher ATP demand and are therefore more susceptible to perturbation of energy production by an uncoupler such as Minerval. Hence, U87-MG cell viability or total ATP is more heavily affected by Minerval as compared with MRC5 cells (Figure 4). However, if we make a more judicious comparison between the lung cancer cell line A549 and the non-malignant lung cell line MRC5, the picture becomes more complicated. A549 and MRC5 cells, perhaps surprisingly, have similar basal OCRs (∼1.5 pmol O_2_ reduced/min/nucleus, Figure 1B,C, upper panels). Therefore, apparently they have similar ATP demands even though one of them is a cancer cell line, which theoretically should have higher ATP demand, and the other is not. It appears that only Minerval triggers the higher energy demand in the A549 cell line as compared with its non-cancer lung derived counterpart MRC5 cell line.

After administration of Minerval, glycolysis was up-modulated only in A549 and not in MRC5 cells (cf Figure 1B,C). The metabolic potential (FCCP stress induced-activation of energy transformation pathways, such as respiration and glycolysis) of both A549 and MRC5 (and also U87-MG) cells relied on glycolysis and not on respiration (Figure 3, compare relative rises in OCR vs ECAR in control cells), i.e., under FCCP stress ECAR, and not OCR, was up-modulated. Therefore, once ATP demand rises distinctively in A549 (and not MRC5) cells by Minerval, they up-modulate glycolysis, not compromised by Minerval as respiration is, in an attempt to salvage ATP.

Within cancer cell lines, there appears to be a difference between the glioblastoma cell line U87-MG and the lung carcinoma cell line A549. Minerval seems more cytotoxic to A549 than to U87-MG. This difference is most apparent if the energy phenotypes of the two cell lines are compared (Figure 3). Minerval decreased overall energy status (both basal and stressed) in A549 cells (Figure 3B). This effect, manifested by a decrease in distance from the upward right corner, is not shared by U87-MG cells, in which the energy status is rather increased by Minerval, as manifested by an upward right hand shift in the energy diagram (Figure 3A). These overall energy data suggest that Minerval was more toxic to A549 cells than it was to the U87-MG cells. This conclusion is further supported by three observations: (1) ATP levels were slightly more reduced by Minerval in A549 (Figure 4B, 37% of control), as compared with U87-MG cells (Figure 4A, 44% of control); (2) Both OCR and ECAR (glycolysis) are compromised by Minerval in A549 cells (Figure 3B), whereas only glycolysis is compromised in U87-MG cells (Figure 3A). The glycolysis compensation damage in A549 cells can be revealed by the fact that the glycolytic up-modulation is less pronounced and does not increase with Minerval exposure time (Figures 1B and 3B); (3) At 72 h, Minerval rendered A549 cells so sensitive to the uncoupler (FCCP) insult that their OCR was even reduced (Figures 1B and 2C), rather than being facilitated as it was expected to be, and indeed was after 72 h Minerval exposure, in U87-MG cells (Figures 1A and 2C). (4) IC_50_ values for Minerval were lower in A549 (ca. 90 μM) than in U87-MG (ca. 400 μM) cells (PVE, submitted). As the glycolytic compensatory response as a whole in A549 cells was significantly less pronounced than in U87-MG cells, some of the relative resilience of U87-MG cells to Minerval (as compared with A549 cells) may be related to their higher capacity to compensate for the respiratory holdback by glycolytic up-regulation. However, at longer exposures to Minerval, this capacity is eventually dwindled. Our observations that A549 cells are more sensitive to Minerval than U87-MG cells suggest that in the clinical settings, where Minerval has only been tested in glioblastoma (and not on any other type of cancer), there is more room for improvement in glioblastoma than in lung cancer. We will discuss this possibility in the next section.

### Potential therapeutic Achilles heels of Minerval

The different toxic efficacies of Minerval toward the two cancer cell lines studied—U87-MG (glioblastoma) and A549 (lung carcinoma)—suggest that the selective cancer toxicity of Minerval has some weak spots that can be improved. As described above, in contrast with A549 cells showing constant and less pronounced up-regulation of glycolysis following Minerval, in U87-MG cells at long enough exposure to Minerval (72 h), the ability to compensate reduced coupling efficiency with up-modulated glycolysis is compromised. This phenomenon in U87-MG cells is manifested both in Figures 1A and 3A. Figure 1A (lower panel) shows it by the lower increase in ECAR following longer Minerval exposure, and Figure 3A shows it by the lower ECAR metabolic potential, or ability to meet an energy demand via up-modulation of glycolysis, at high Minerval exposure times. This reduced glycolytic compensation means that the efficacy of Minerval (i.e*.* increase in selective toxicity) might be improved with glycolytic inhibitors (e.g. the FDA approved 2-deoxyglucose, or 3-bromopyruvate). Further inhibition of glycolysis, especially if Minerval dosage used for treating glioblastoma patients is equivalent to cell exposure times of below 72 h, may impede the recovery of cancer cells from Minerval and thus make the drug more efficient. In addition to compromised glycolytic compensation, another possible Achilles heel of cells treated with Minerval is ER stress (Figure 9 and [12]). These Achilles’ heels can be exploited therapeutically. Improvements of Minerval cancer toxicity may be obtained if Minerval is co-administrated in cocktails with inhibitors of glycolysis. Nevertheless, Minerval co-administration with ER stress or unfolded protein response inducers would probably be too harsh for most patients. The expected higher cancer toxicity of Minerval in such cocktails might be evaluated in the future in non-responding or relapsing-remitting patients.

## Abbreviations

ECAR: extracellular acidification rate
ER: endoplasmic reticulum
FCCP: carbonyl cyanide-4-(trifluoromethoxy)phenylhydrazone
MLT: membrane lipid therapy
MMP: mitochondrial membrane potential
OCR: oxygen consumption rate
OxPhos: oxidative phosphorylation
ROS: reactive oxygen species
